# Neurocognitive Interventions Informed by Cognitive–Behavioral Therapy (CBT) Principles and Physical Exercise for Complex Regional Pain Syndrome: A Systematic Review

**DOI:** 10.3390/jcm14196820

**Published:** 2025-09-26

**Authors:** Leidy Tatiana Ordoñez-Mora, Daihana Stefany Quintero-López, Marco Antonio Morales-Osorio, Juan Fernando Gómez-Gómez, Giovanna Patricia Rivas-Tafurt, María Fernanda Serna-Orozco

**Affiliations:** 1 Physiotherapy Program, Department of Health, Universidad Santiago de Cali, Santiago de Cali 760035, Colombia; dahiana.quintero00@usc.edu.co (D.S.Q.-L.); maria.serna05@usc.edu.co (M.F.S.-O.); 2 Carrera de Kinesiología, Escuela de Kinesiología, Facultad de Ciencias de la Rehabilitación y Calidad de Vida, Universidad San Sebastián, Lientur 1457, Concepción 4030000, Chile; marco.morales@uss.cl; 3 Internal Medicine Specialization Program, Department of Health, Universidad Santiago de Cali, Santiago de Cali 760035, Colombia; juan.gomez51@usc.edu.co (J.F.G.-G.); giovanna.rivas@clinicadeoccidente.com (G.P.R.-T.); 4 Research and Education Group GIEDCO, Clínica de Occidente S.A., Santiago de Cali 760046, Colombia

**Keywords:** complex regional pain syndrome, pain, physical function, activities of daily living, neurocognitive interventions informed by CBT principles

## Abstract

**Background:** Complex Regional Pain Syndrome (CRPS) is a chronic pain condition that usually affects a limb following injury or surgery and is characterized by severe pain accompanied by sensory, motor, autonomic, and trophic disturbances. **Methods:** This systematic review aimed to synthesize the available evidence on the effectiveness of physical exercise and neurocognitive interventions grounded in cognitive–behavioral therapy (CBT) principles for the management of CRPS. A comprehensive search was conducted in Medline (via Ovid), LILACS, ScienceDirect, PEDro, OTseeker, and the Cochrane Central Register of Controlled Trials (CENTRAL). Eligible studies included clinical trials, cohort studies, and cross-sectional studies, whereas case reports, pediatric populations, and animal studies were excluded. Fifteen studies met the inclusion criteria. **Results:** The findings indicated that aerobic exercise was consistently associated with pain reduction and functional improvement. Neurocognitive interventions informed by CBT principles, such as mirror therapy and graded exposure, also demonstrated efficacy in decreasing pain and enhancing functional independence. Most studies supported the effectiveness of these approaches in the management of CRPS. Overall, both physical exercise and neurocognitive interventions grounded in CBT principles produced positive effects on pain modulation, physical function, and daily activity performance. **Conclusions:** These findings highlight the therapeutic potential of combining physical and psychologically informed interventions for the treatment of CRPS.

## 1. Introduction

Complex Regional Pain Syndrome (CRPS) is a chronic condition characterized by severe and persistent pain that may affect one or more extremities [1]. The worldwide incidence of CRPS is unknown; however, some epidemiological studies report an incidence rate ranging from 5.46 to 26.2 cases per 100,000 persons per year [2,3]. CRPS occurs most frequently in adults between 40 and 70 years of age; women are more affected than men at a ratio of 3:1, and in children, girls are more frequently affected than boys at a ratio of 9:1 [4]. In adults, the upper extremities are usually more affected than the lower extremities [5], whereas in children, the lower extremities are typically more affected than the upper extremities [6].

The pathogenesis of CRPS is multifactorial, most commonly associated with fractures or surgery, but it can also occur after spinal cord, nerve, or ligament injury, and in some cases spontaneously. Peripheral sensitization, autonomic nervous system dysregulation, and immune dysfunction are thought to contribute to the development of CRPS [7]. It is classified into two types: type I (reflex sympathetic dystrophy) occurs without confirmed peripheral nerve injury, and type II (causalgia) occurs in the presence of such injury [8]. The main symptoms include allodynia or hyperalgesia disproportionate to the precipitating event, as well as skin changes, sudomotor dysfunction, or edema [9].

The treatment of CRPS is multidisciplinary and includes pharmacological management, sympathetic nerve blocks, psychotherapy, occupational therapy, physiotherapy, and complementary therapies [10]. Among these strategies, clinical guidelines highlight physiotherapy as a key approach to controlling pain and disability. This includes therapeutic exercise and techniques based on neurocognitive rehabilitation (such as mirror therapy or graded motor imagery), which integrate cognitive and behavioral principles to address maladaptive pain perception and motor function [11,12,13]. While these are informed by psychological approaches, they do not constitute formal Cognitive–Behavioral Therapy (CBT) as delivered by mental health professionals.

CBT is a structured, time-limited intervention aimed at improving emotional and functional status by identifying and modifying maladaptive thoughts and behaviors [14]. Although traditionally based on techniques such as cognitive restructuring, thought records, and behavioral exposure, in the context of CRPS, complementary interventions have emerged. While not directly derived from CBT, these methods share cognitive and behavioral principles and are integrated into a comprehensive therapeutic approach. This review therefore also considers techniques such as mirror therapy [15], graded motor imagery [16,17], prism adaptation [18,19], virtual reality-mediated interventions [20], and pain exposure physical therapy [17,21], as well as any intervention grounded in cognitive–behavioral principles, due to their utility in modifying pain perception, movement patterns, and coping strategies.

Physical exercise, on the other hand, has proven to be an effective and safe therapeutic tool in the management of CRPS. Clinical guidelines recommend it as a fundamental component of the multimodal approach. Various studies indicate that interventions such as progressive mobilization, aerobic exercise, and aquatic therapy can help reduce pain, improve physical function, and decrease edema in patients with CRPS type I [22]. Although further research is needed to determine optimal protocols, current evidence strongly supports the inclusion of exercise as a key element of rehabilitation.

Despite the range of available interventions, evidence on the rehabilitative treatment of CRPS remains limited. It is still unclear which strategies are most effective in reducing pain and disability, or whether their combination with conventional therapies yields superior outcomes. Therefore, the purpose of this review is to examine the effectiveness of physical exercise and neurocognitive interventions informed by cognitive and behavioral principles in the treatment of patients with CRPS, in order to support evidence-based clinical decision-making.

## 2. Materials and Methods

This systematic review was conducted in accordance with the PRISMA 2020 guidelines [23], as detailed in the Supplementary Materials. The protocol was registered in the Prospective International Register of Systematic Reviews (PROSPERO) under registration number CRD4202020209914.

### 2.1. Selection Criteria

The following research question (RQ) was established:

- Population: adult patients (≥18 years) diagnosed with complex regional pain syndrome.
- Intervention: physical exercise and cognitive–behavioral interventions.
- Comparison: Conventional therapy or usual care, including hydrotherapy, early stimulation (e.g., TENS), physical modalities, occupational therapy, and facilitation techniques, without neurocognitive elements.
- Outcomes: Pain: Measured using the Numerical Rating Scale (NRS), Visual Analog Scale (VAS), or McGill Pain Questionnaire.; Physical function: Assessed through passive range of motion (via goniometry) and strength (using a hand dynamometer); and Functional Outcomes or Disability Related to Daily Activities: Evaluated using instruments such as the Patient-Rated Wrist/Hand Evaluation (PRWHE), QuickDASH, the 5-item version of the EQ-5D (EQ-5D-5L), the Oswestry Low Back Pain Disability Questionnaire, and the Roland-Morris Disability Questionnaire.

Included studies:

We included experimental, quasi-experimental, cohort, case–control, and cross-sectional designs involving adult patients diagnosed with CRPS type I or II, associated with neurological or musculoskeletal conditions.

Exclusion criteria:

We excluded case reports, studies involving pediatric populations or animal models, and research focused exclusively on pharmacological or surgical interventions without including physical exercise or cognitive–behavioral therapy.

### 2.2. Source of Information and Strategy Search

A comprehensive literature search was conducted using Medical Subject Headings (MeSH), DeCS terms, and related keywords. The following databases were searched from their inception to 31 December 2024: MEDLINE (via Ovid), ScienceDirect, LILACS, PEDro, OTseeker, and the Cochrane Central Register of Controlled Trials (CENTRAL). No language or publication date restrictions were applied. The search strategy included terms such as “Complex Regional Pain Syndromes”, “Physical Therapy”, “Cognitive-Behavioral Therapy”, “Physical Therapy Modalities”, “Exercise”, “Mirror Therapy”, “Motor Imagery”, “Range of Motion, Articular”, “Activities of Daily Living”, and “Motor Function”, applied to titles and abstracts. The complete search strategy is available in the PROSPERO protocol and detailed in Appendix A.

### 2.3. Selection of Articles

The study selection process began with a calibration phase to ensure consistency between reviewers during screening. References were managed using EndNote, and a manual filtering process was conducted in Microsoft Excel. Two reviewers independently screened titles and abstracts, followed by full-text review according to pre-defined inclusion and exclusion criteria. Disagreements were resolved through consensus; if unresolved, a third reviewer acted as arbiter. A pilot calibration phase was conducted to ensure consistency. Data were independently extracted from the included studies by the same reviewers using a standardized extraction form.

### 2.4. Process for Collecting Information

Two expert physiotherapist reviewers independently extracted data from each study using a standardized Excel matrix. The extracted information included study design, geographic location, authors and year of publication, title, objectives, methodology, sample size and participant age, CRPS type, inclusion and exclusion criteria, number of participants, description of the intervention and comparison groups, and outcomes related to pain and functional performance or disability in daily activities, along with the corresponding measurement instruments.

### 2.5. Article Quality Rating and Risk of Bias

The PEDro scale [24] was used to assess methodological quality of randomized controlled trials, and the MINORS tool was applied to non-randomized studies. The results of these evaluations were summarized narratively and visualized using figures that illustrate the fulfilment of each item from the PEDro and MINORS scales. High risk of bias related to allocation and blinding was noted across several studies and is discussed in the results and limitations.

For randomized trials, the PEDro scale evaluates criteria such as randomization, allocation concealment, baseline comparability, blinding of participants, therapists, and assessors, outcome data completeness (≥85% for at least one key outcome), intention-to-treat analysis, between-group statistical comparisons, and reporting of point estimates and measures of variability. Studies scoring 6 or higher on the PEDro scale were included in the review.

For observational and non-randomized studies, methodological quality and risk of bias were assessed independently and blindly using the MINORS (Methodological Index for Non-Randomized Studies) scale. This tool evaluates elements such as a clearly stated objective, inclusion of consecutive patients, prospective data collection, appropriate analysis, reporting of data loss, and baseline equivalence between groups. A minimum score of 11 on the MINORS scale [25] was required for study inclusion.

### 2.6. Data Analysis and Results Synthesis

Quantitative data were extracted for descriptive analysis. Due to the high heterogeneity in interventions (e.g., Mirror Therapy, GMI, Prism Adaptation) and outcomes, the results are synthesized narratively and grouped by type of intervention to aid interpretation. A meta-analysis was not conducted due to substantial clinical and methodological heterogeneity, as well as variability in the assessment methods, which prevented a reliable and objective synthesis of the results.

## 3. Results

### 3.1. Selection of Studies

**Figure 1:**
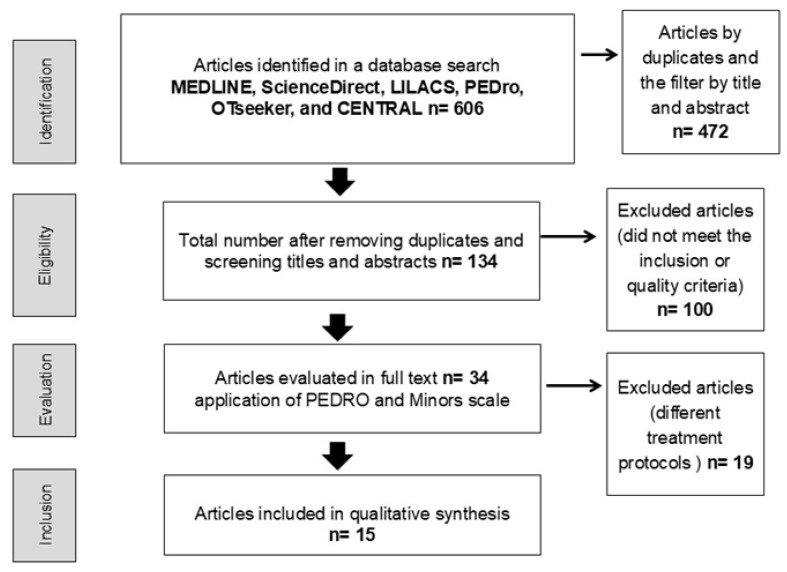
PRISMA 2020 flow diagram illustrating the study selection process, adapted from the official PRISMA template (www.prisma-statement.org accessed on 10 March 2025).

The initial search identified a total of 606 studies. After removing duplicates, 317 studies were screened, and 34 were selected for full-text review. Nineteen studies were excluded because they involved treatment protocols unrelated to the intervention or outcome of interest (see Appendix B for a complete list of excluded studies and reasons). The remaining 15 studies were included in this review: Moseley et al., 2004 [26], Moseley et al., 2006 [27], Cacchio et al., 2009 [28], Lagueux et al., 2012 [29], Pervane et al., 2015 [30], Barnhoorn et al., 2015 [31], Topcuoglu et al., 2015 [32], Barnhoorn et al., 2015 [33], Christophe et al., 2016 [34], Hollander et al., 2016 [35], Schmid et al., 2017 [36], and Halicka et al., 2021 [19], Machač et al., 2024 [37], Lewis et al., 2021 [38], Saha et al., 2021 [39] (see Figure 1).

### 3.2. Characteristics of Excluded Studies

Excluded articles were not related to the intervention or outcome of interest. Additionally, letters to the editor, systematic reviews/narratives, pharmacologic intervention protocols, animal studies, and studies with pediatric populations were excluded.

### 3.3. Characteristics of the Included Studies

**Figure 2:**
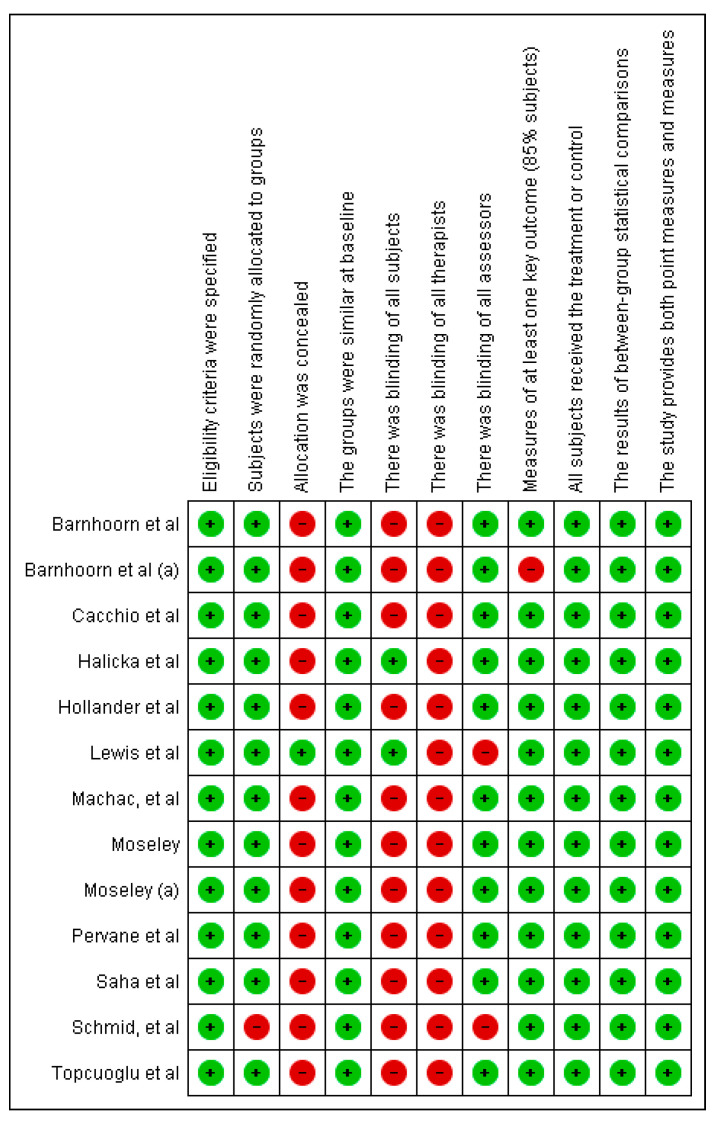
Methodological quality assessment of randomized controlled trials using the PEDro scale. Green circles (+) indicate that the criterion was fulfilled, while red circles (−) indicate that it was not fulfilled. References: Moseley et al., 2004 [26]; Moseley et al., 2006 [27]; Cacchio et al., 2009 [28]; Pervane et al., 2015 [30]; Barnhoorn et al., 2015 [31]; Topcuoglu et al., 2015 [32]; Barnhoorn et al., 2015 [33]; Hollander et al., 2016 [35]; Schmid et al., 2017 [36]; Halicka et al., 2021 [19]; Machač et al., 2024 [37]; Lewis et al., 2021 [38]; Saha et al., 2021 [39].

**Figure 3:**
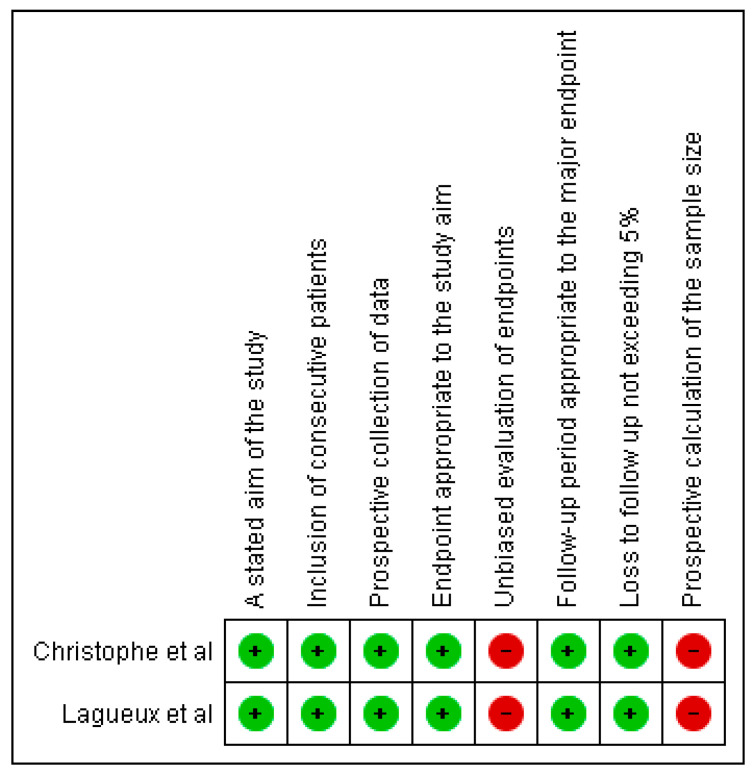
Methodological quality assessment of non-randomized studies using the MINORS tool. Green circles (+) indicate that the criterion was fulfilled, while red circles (−) indicate that it was not fulfilled. References: Christophe et al., 2016 [34]; Lagueux et al., 2012 [29].

The 15 studies included in this review were published between 2004 and 2024. All studies included participants of both sexes, with sample sizes ranging from seven participants [29,34] to 56 participants [31]. Four studies evaluated interventions in stroke patients [28,30,32,39]. One study [26] focused on adults with wrist fractures, while the remaining studies did not specify the type of injury or the underlying causes associated with the occurrence of CRPS (Figure 2 and Figure 3).

Regarding the condition evaluated, three studies included patients with CRPS affecting both upper and lower extremities [31,33,35]. Eleven studies exclusively involved patients with upper extremity CRPS, while only one study [34] included patients with both CRPS types (type I and II). Geographically, most studies were conducted in Europe (12/15), with the remaining studies conducted in North America (1/15), Australia (1/15), and India (1/15) (Table 1).

Quality and risk of bias assessment (PEDro and MINORS).

### 3.4. Studies Included and Type of Intervention

Thirteen of the 15 selected studies were randomized controlled trials (RCTs). Nine of these reported follow-up data after the intervention [19,26,27,28,33,35,38,39]. The remaining two studies were a pilot study [36], an intervention study [34], and a pretest–posttest study [29], all with no comparison group. The interventions evaluated in these studies included aerobic exercise [32]; motor imagery [26,27,29]; prism adaptation [19,34,35]; pain exposure therapy [33,35]; sensorimotor training [36]; and mirror therapy [28,30,37,39]. Only one study [38] involved visual manipulation using virtual reality (Table 2).

To enhance interpretability, interventions were categorized into subgroups: (1) Mirror Therapy, (2) Graded Motor Imagery, (3) Prism Adaptation, (4) Pain Exposure Therapy, (5) Sensorimotor Training, and (6) Aerobic Exercise. This structure allows comparison of effects within and across modalities (Table 3, Table 4 and Table 5).

Certainty analysis: The GRADE analysis showed that mirror therapy (MT) and pain exposure physical therapy (PEPT) reached a moderate level of certainty across pain, physical function, and activities of daily living outcomes. MT, supported by four RCTs, demonstrated consistent improvements in pain reduction, functional performance, and daily activity measures. PEPT, evaluated in three RCTs, also showed beneficial effects on range of motion and disability, with some variability in strength outcomes. In contrast, graded motor imagery (GMI), prism adaptation, virtual reality, aerobic exercise, and sensorimotor training were supported by single or small pilot studies, with methodological limitations and imprecision, leading to low or very low certainty ratings.

### 3.5. Effects of Physical Exercise on Pain, Physical Function, and Functional Outcomes

Only one included study specifically evaluated the effects of physical exercise in patients with CRPS [32]. This study implemented a four-week aerobic exercise program combined with conventional therapy, conducted five times per week in 30 min sessions. The intervention group showed significant post-treatment reductions in pain, hyperalgesia, and tenderness in the wrist and metacarpophalangeal joints, along with decreased movement-related pain throughout the day compared to the control group. Although no significant differences were observed in physical function as measured by the Brunnstrom stages of the upper limb and hand, the intervention group demonstrated significantly higher scores on the Functional Independence Measure (FIM), including both motor and cognitive domains (Table 2).

### 3.6. Effects of Neurocognitive Interventions Informed by CBT Principles on Pain

All 15 included studies assessed pain levels pre- and post-intervention using various validated scales. The most frequently used was the Visual Analogue Scale (VAS) (*n* = 10), followed by the Neuropathic Pain Scale (*n* = 4) and the Pain Inventory Index (*n* = 1). Overall, most studies reported significant reductions in pain following the intervention, with statistically significant differences compared to control groups in all but two studies [19,33].

One study observed a reduction in pain in patients with CRPS affecting both upper and lower limbs after pain exposure physical therapy; however, intention-to-treat analysis revealed no significant differences between groups [31]. In contrast, ref. [35] reported a statistically significant reduction in pain intensity immediately post-intervention and at 6-month follow-up in a cohort receiving pain exposure therapy compared to conventional care. Sensorimotor training also yielded positive results. One study [36] implemented a two-week protocol involving a Braille-like haptic task in various training modes and found that pain reduction correlated with the total training duration.

Three studies evaluated graded motor imagery in patients with upper limb CRPS. Refs. [26,27] found a significant reduction in pain compared to conventional therapy, with the most notable improvement occurring during the mirror movement phase. These effects persisted from 6 weeks to 6 months post-treatment. Similarly, ref. [29], despite lacking a control group, reported significant pain reductions, especially during the later phases of therapy involving mirror movements of the unaffected limb.

Mirror therapy interventions demonstrated consistent benefits. Ref. [37] found a significant pain reduction (*p* < 0.001) in CRPS type I patients, sustained up to one-month post-intervention. Refs. [28,30] also demonstrated that the combination of mirror therapy with conventional therapy produced significant pain improvement in patients with upper limb CRPS, with a treatment duration of four weeks. Both studies reported significant pain reductions and observed lasting pain reduction for up to six months. Ref. [39] showed that mirror therapy led to a significant pain reduction from 6.07 ± 1.58 to 3.93 ± 1.39 (*p* < 0.05) on the Numerical Pain Rating Scale (NPRS), with more pronounced effects than in the control group.

Regarding visual-perceptual interventions, ref. [19] found no significant pain reduction using prism adaptation therapy. In contrast, ref. [34] reported a 36% decrease in baseline pain following the same intervention. Ref. [38] demonstrated a significant pain reduction using virtual reality visual illusions (*p* = 0.037), while the control group showed no change (*p* > 0.05), suggesting that visual-based interventions may offer innovative pain management strategies for CRPS (Table 2).

### 3.7. Effects of Neurocognitive Interventions Informed by CBT Principles on Physical Function

Studies evaluated the impact of cognitive–behavioral interventions on physical function. Refs. [28,30] reported significant improvements in motor function in post-stroke CRPS patients following mirror therapy. Functional gains were measured using the Fugl–Meyer Assessment in the former and the Wolf Motor Function Test in the latter. Ref. [31] found a significant increase in active range of motion following pain exposure therapy compared to controls. Although muscle strength improved progressively in the intervention group, the between-group difference was not statistically significant. Ref. [29] reported improved grip strength in the affected limb during the acute phase of graded motor imagery therapy (Table 2).

### 3.8. Effects of Neurocognitive Interventions Informed by CBT Principles on Functional Outcomes or Disability Related to Daily Activities

Eight studies reported outcomes related to functional outcomes or disability associated with daily activities [28,29,30,31,33,34,35,39]. Mirror therapy appeared particularly beneficial. Refs. [30,39] found significant improvements in FIM motor scores following mirror therapy in patients with upper limb CRPS. Similarly, ref. [28] observed significant improvement in the Motor Activity Log (MAL) score. In all cases, mirror therapy contributed to enhanced functional independence.

Three studies examined pain exposure therapy in relation to disability associated with daily activities. Ref. [31] found no significant differences in EQ-5D index scores between groups, while another study by the same authors [33] reported a significant reduction in disability on the Pain Disability Index. Ref. [35] observed reductions in upper and lower extremity disability at six-month follow-up, measured via the Radboud Ability Questionnaire and the Ability to Walk Questionnaire, respectively. Results for other interventions were mixed. Ref. [29] reported no significant change in perceived upper limb function on the Disabilities of the Arm, Shoulder, and Hand (DASH) questionnaire. In contrast, ref. [34] reported a 10-point improvement on the Sickness Impact Profile (SIP) following prism adaptation therapy, suggesting potential for functional gains in daily living activities.

## 4. Discussion

This systematic review indicates that CBT-informed neurocognitive interventions—especially mirror therapy and graded motor imagery (GMI)—and, to a lesser extent, aerobic exercise, may reduce pain and improve physical function and daily activities in patients with CRPS when added to conventional care. Mirror therapy showed the most consistent benefits in upper-limb CRPS [28,30]. Evidence for aerobic exercise is preliminary and requires further study.

GMI may reduce pain when integrated with standard care; however, effects on physical function and daily activities remain uncertain, with only one study assessing these outcomes [28].

Findings for prism adaptation and pain exposure therapy were mixed. Some trials reported pain reductions, whereas others found no clear differences versus controls [19,31,33,34]. Two studies of pain exposure therapy reported improvements in disability [31,33], suggesting functional benefits. Prior literature aligns with these results. Smart et al. (2022) reviewed electrotherapy, cortically directed rehabilitation, electroacupuncture, and exposure-based therapies, noting that most studies were small and at high risk of bias; no intervention—including multimodal physical therapy—showed clear superiority for pain or disability [13].

Harden et al. (2022) likewise reported predominantly low-to-moderate methodological quality, largely due to small samples inherent to the rarity of CRPS, but highlighted positive effects of strengthening, body-awareness training, sensorimotor re-education, massage, and myofascial release [40]. In our review, mirror therapy emerged as the most consistently beneficial neurocognitive approach for upper-limb CRPS, whereas evidence for aerobic exercise—although compatible with previous reports—remains preliminary [41,42].

Evidence from Méndez et al. (2021) supports pain reductions following mirror therapy and motor imagery [43]. Schubert et al. (2021) found GMI particularly effective in acute CRPS, with associated improvements in cortical representation [44]. These findings support integrating CBT-informed neurocognitive rehabilitation into CRPS management.

CRPS requires multidisciplinary care due to its complex and persistent symptomatology. Reviews by Johnson et al. (2022) and Kraft et al. (2021) show that pain and motor dysfunction frequently persist long-term; 51–89% of patients continue to experience symptoms beyond 12 months, contributing to substantial disability [45,46].

The certainty of evidence (GRADE) ranged from very low to moderate, depending on intervention and outcome. Mirror therapy achieved moderate certainty for pain and functional improvement, limited by small samples and potential publication bias. Certainty for GMI was very low due to risk of bias, heterogeneity, and imprecision from small samples. Prism adaptation and virtual reality showed promising but low-certainty effects. Aerobic exercise and sensorimotor training were supported by very-low-certainty evidence. Overall, effects trend favorably toward neurocognitive interventions and exercise, but higher-quality studies are needed to strengthen confidence.

### 4.1. Strengths and Limitations

This review synthesizes evidence on physical exercise and CBT-informed neurocognitive interventions—mirror therapy and GMI—delivered by physical therapists for individuals with CRPS. These approaches, when added to conventional therapy, show potential for clinical implementation; however, more high-quality trials are required to confirm effectiveness.

Limitations include the small number of CRPS-specific studies, methodological heterogeneity in populations and protocols, and high risk of bias related to randomization, allocation concealment, and blinding. Outcome measures varied widely (e.g., VAS, NRS, DASH, WMFT, EQ-5D), preventing meaningful pooling. Intervention protocols differed in duration, frequency, content, and co-interventions, further limiting meta-analysis and risking misleading estimates. We therefore used a narrative synthesis with GRADE tables to appraise the evidence. These factors reduce confidence in current estimates and underscore the need for rigorous trials with standardized outcomes and clearer reporting.

Most studies did not report sufficient data (means, standard deviations, or adjusted estimates) to calculate standardized effect sizes with 95% confidence intervals. As a result, outcomes were often limited to *p*-values or mean changes, hampering cross-study comparisons. Future trials should consistently report standardized effect sizes and confidence intervals to improve comparability and clinical interpretation.

Formal subgroup or moderator analyses were not feasible due to few studies per category and inconsistent reporting of intervention duration, provider characteristics, and patient subgroups (e.g., post-stroke vs. post-fracture). Although mirror therapy appeared more consistent in upper-limb CRPS and GMI was mainly applied in post-stroke populations, the evidence is insufficient to draw firm conclusions. Standardized reporting is needed to enable stratified analyses and provide clearer clinical guidance.

Training is another gap. Few studies described how therapists were prepared to deliver GMI, mirror therapy, or pain exposure therapy. Future research should standardize training protocols and evaluate how therapist competence influences outcomes.

Finally, interdisciplinary collaboration is essential. Combining physiotherapy with explicit psychoeducation and mental-health support may enhance adherence and address the biopsychosocial complexity of CRPS.

### 4.2. Research Priorities

Future work should address methodological gaps through adequately powered, multicenter randomized controlled trials with long-term follow-up. Direct head-to-head comparisons among neurocognitive interventions (e.g., mirror therapy, GMI, prism adaptation, exposure therapy) and versus conventional physiotherapy are needed to determine relative efficacy. Factorial designs combining modalities (e.g., aerobic exercise plus mirror therapy) should explore potential synergy. Trials should incorporate mechanistic outcomes—quantitative sensory testing, neuroimaging, and psychological assessments (e.g., fear-avoidance, catastrophizing)—to link clinical change with underlying processes. Protocols should be standardized (at least ~12 weeks, two sessions per week) to improve comparability and inform guidelines.

### 4.3. Public Health Significance

CRPS imposes a substantial burden on patients and health systems. Non-pharmacological guidelines increasingly recognize the role of physical exercise as a modulatory intervention for this population. Future studies should adopt standardized outcomes: pain (Visual Analogue Scale), motor function (Fugl–Meyer Assessment or Wolf Motor Function Test), strength (dynamometry), and activities of daily living (DASH, Functional Independence Measure). Protocols should last ≥12 weeks with at least two sessions per week, and include well-defined control groups to support robust, generalizable recommendations.

## 5. Conclusions

Physical exercise and CBT-informed neurocognitive interventions show potential benefits for pain, physical function, and daily activities in CRPS. The evidence base remains limited, heterogeneous, and methodologically variable, highlighting the need for high-quality, multidisciplinary trials.

## Figures and Tables

**Table 1:** General characteristics of the included studies.

Studio	Country	Type of Study	Type of Cprs/Body Segment	Sample	Intervention	Sample Genre	Comparison	Sample Genre	Results
Moseley et al., 2004 [26]	Australia	RCT/12 months of follow-up	CPRSt/upper extremity	Adult patients after wrist fracture	Motor Imaging Program	7:5 women/2 men	CT	6:4 women/2 men	The treatment group showed a effect size of 25 points on the neuropathic pain scale.
Moseley et al., 2006 [27]	England	RCT/6 months follow-up	CPRSt1/upper extremity	Adult patient	Motor imagery program	25	CT	25	A decrease in pain between pre- and post-treatment (100 mm visual analog scale) of 23.4 mm (from 16.2 to 30.4 mm) for the motor imagery group and 10.5 mm (from 1.9 to 19.2 mm) for the control group.
Cacchio et al., 2009 [28]	Rome	RCT/6-month follow-up	CPRSt1/upper extremity	Stroke patients	MT + CT	24:13 women/11 men	CT	24:13 women/11 men	Reduction in visual analog scale score of pain at rest, on movement and brush-induced tactile allodynia in the mirror group (*p* < 0.001).
Lagueux et al., 2012 [29]	Canada	Pre-experimental pretest–posttest design	CPRSt1/upper extremity	Adult patients	GMI	7:6 women/1 man	No comparison		Decrease in pain experienced in the last 7 days (visual analog scale; *p* = 0.046), improvement of the affected limb. grip strength (*p* = 0.042), and the patient’s overall impression of change (*p* = 0.015).
Pervane et al., 2015 [30]	Turkey	RCT	CPRSt1/upper extremity	Stroke patients	MT + CT	15:6 women/9 men	CT	15:7 women/8 men	Both groups had significant improvements in pain and motor function, but the MT group improved more (*p* < 0.001, and *p* = 0.03, respectively).
Barnhoorn et al., 2015 [31]	Netherlands	RCT/3, 6 and 9 month follow-ups	CPRSt1/upper and lower extremities	Adult patients	PEPT	35:29 women/6 men	CT	21:16 women/5 men	The experimental group had a significantly greater decrease in disability of 7.77 points (95% CI 1.09 to 14.45) and pain of 1.83 points (95% CI 0.44 to 3.23) over 9 months than the control group.
Topcuoglu et al., 2015 [32]	Turkey	RCT	CPRSt1/upper extremity	Stroke patients	Aerobic exercise + CT	20:9 women/11 men	CT	20:9 women/11 men	The exercise group showed less daytime pain and pain on movement in the shoulder (*p* = 0.009, *p* = 0.012) and less daytime pain, nighttime pain and pain on movement in the hand (*p* = 0.041, *p* = 0.001).
Barnhoorn et al., 2015 [33]	Netherlands	RCT/9 months of follow-up	CPRSt1/Upper and lower extremity	Adult patients	PEPT	28:24 women/4 men	CT	28:21 women/7 men	Decreased pain and improved active range of motion and % muscle strength in the PEPT group compared to CT, but the difference between groups was only significant in active range of motion (*p* = 0.02).
Christoph e et al., 2016 [34]	France	Intervention study	CPRSt1, CPRSt2/upper extremity	Adult patient	PA	7:6 women/1 man	No comparison	-	Improvement in quality of life after the 10-point intervention on the SIP scale. The preglobal score was 38.7 ± 5.76 points (mean ± SEM), while the postglobal score was only 28.6 ± 4.64 points.
Hollander et al., 2016 [35]	Netherlands	RCT/6-month follow-up	CPRSt1/upper and lower extremities	Adult patients	In vivo exposure (EXP)	23:18 women/5 men	Pain-dependent treatment	23:19 women/4 men	EXP was superior to TAU in the reduction of upper and lower extremity disability from pretreatment to post-treatment and from pre-treatment to 6-month follow-up (*p* < 0.001).
Schmid et al., 2017 [36]	Germany	Pilot study	CPRSt1/upper extremity	Adult patients	Sensorimotor training	10:3 men/7 women	No comparison	-	Patients showed a significant reduction in pain after the 2-week training period. Changes in pre-post VAS had a mean of 1.0 ± 1.19 (mean ± SD) with a range between −1 and +3, which represented a statistically significant decrease(t(9) = 2.748, *p* = 0.023).
Halicka et al., 2021 [19]	England	RCT/3 and 6 months follow-up	CPRSt1/upper extremity	Adult patients	PA	23:19 women/4 men	CT	26:22 women/4 men	There is no evidence that primary or secondary outcomes differed between the prism adaptation and sham treatment groups when assessed at any of the post-treatment time points.
Machač et al., 2024 [37]	Czech Republic	RCT	CPRSt1/upper extremity	Adult patient	MT	13	No treatment	14	Changes in the Visual Analog Scale (VAS) pre-post had a mean of −2.9 ± 1.4 (mean ± SD) with a range between −1.5 and −5.3, which represented a statistically significant decrease = 3.82, *p* = 0.002).
Lewis et al., 2021 [38]	Reuno United	RCT	CPRSt1/upper extremity	Adult patient	MVR	23	No actual image manipulation	22	In the experimental group, changes in NRS were from 5.6 ± 3.0 to 4.4 ± 2.5 (*p* = 0.037), while in the control group no significant changes were observed (*p* > 0.05). In addition, body perception improved in the experimental group (38.7 ± 5.76 to 28.6 ± 4.64, *p* = 0.036), but not in the control group (*p* = 0.056).
Saha et al., 2021 [39]	India	RCT	CPRSt1/upper extremity	Adult patient	MT	15	CT	15	The results of the study showed that mirror therapy combined with postictus rehabilitation produced a significant reduction in pain and an improvement in functional independence compared to non-mirror rehabilitation.

CPRSt1: Complex Regional Pain Syndrome type 1; CPRSt2: Complex Regional Pain Syndrome type 2; MT: mirror therapy; CT: Conventional therapy (no neurocognitive elements); PEPT: Pain Exposure Physical Therapy; PA: Adaptive Prism; SIP Scale: Disease Impact Profile Scale. MVR: Mediated Virtual Reality; GMI: Graded motor imagery.

**Table 2:** Outcome measures in pain, physical function and activities of daily living of the included studies.

Studio	Intervention (Time/Type)	Measure	Scale	Intervention	Comparison	*p*-Value
Moseley et al., 2004 [26]	Motor imagery program 6 weeks	Pain	NPS	Baseline: Total NPS: 46 (4.2) NPS intensity item: 6.6 (0.5) Post-treatment 6 weeks: Total NPS: 20 (10.1–29.9) Intensity point NPS: 3 (2.6–5.4) Post-treatment 12 weeks: Total NPS: 20 (10.1–29.9) Intensity point NPS: 3 (2.6–5.4)	Baseline: Total NPS: 44 (4.3) Intensity item NPS: 6.0 (1.1) Post-treatment: No change	<0.001
Moseley et al., 2006 [27]	Motor imagery program 2 weeks	Pain	VAS	2.34 (1.2 a 3.04) 6 months: 3.21 (2.38–4.03)	1.05 (0.9–1.92) 6 months: 1.16 (0.24–2.07)	0.002
Cacchio et al., 2009 [28]	Mirror therapy 4 weeks	Pain	VAS	Pain at rest: Pre-treatment 7.6 ± 1.2 Post-treatment 4.3 ± 2.5 Pain on movement: Pretreatment 8.7 ± 0.6 Post-treatment 5.1 ± 2.6	Pain at rest: Pretreatment 7.5 ± 1.1 Post-treatment 7.2 ± 2.2 Pain on movement: Pretreatment 8.3 ± 0.7 Post-treatment 8.2 ± 1.4	0.828 <0.001 0.04 <0.001
		Physical function	WMFT	Pretreatment 3.5 ± 1.2 Post-treatment 1.5 ± 0.7	Pre-treatment 3.6 ± 0.7 Post-treatment 3.4 ± 0.9	0.726 <0.001
		Activities of daily living	MAL	Pretreatment 1.4 ± 0.4 Post-treatment 3.6 ± 1.5	Pretreatment 1.3 ± 0.5 Post-treatment 1.2 ± 0.8	0.468 <0.001
Lagueux et al., 2012 [29]	Graded motor imagery 4–12 weeks	Pain	SF-MPQ/VAS	Start: 4.39 (2.24) Post-treatment: 2.05 (2.33)	No comparison	0.046
		Physical function	Grip strength	Onset: 11.78 (9.02) mmHg Post-treatment: 28.90 (13.10) mmHg	No comparison	0.042
		Activities of daily living	DASH	Home: 40.97 (12.29) Post-treatment: 31.98 (16.97)	No comparison	0.138
Pervane et al., 2015 [30]	Mirror therapy 4 weeks	Pain	VAS	Start: 6 (2–9) Post-treatment: 3 (1–6)	Start: 5 (3–9) Post-treatment: 5 (2–8)	<0.001
		Physical function	FMA	Hand: Baseline: 7 (2–13) Post-treatment: 11 (5–14) Wrist: Baseline: 5 (2–6) Post-treatment: 8 (4–9)	Hand: Baseline: 5 (3–13) Post-treatment: 5 (3–13) Wrist: Baseline: 4 (1–8) Post-treatment: 5 (1–8)	<0.001 <0.001
		Activities of daily living	FIM	Start: 41 (20–83) Post-tratamiento: 44 (20–83)	Home: 32 (14–86) Post-treatment: 39 (15–86)	0.09
Barnhoorn et al., 2015 [31]	Pain exposure physiotherapy 5 sessions	Pain	VAS	Home: 6.23 (2.40) 3 months: 3.97 (2.47) 6 months: 3.90 (2.62) 9 months: 3.24 (2.31)	Home: 7.33 (1.96) 3 months: 6.24 (3.21) 6 months: 5.65 (3.42) 9 months: 5.89 (3.16)	0.01
		Activities of daily living	PDI	Base: 33.91 (13.00) 3 months: 19.87 (13.56) 6 months: 12.48 (11.96) 9 months: 10.40 (9.54)	Base: 37.17 (13.06) 3 months: 28.18 (16.38) 6 months: 22.58 (16.23) 9 months: 23.48 (18.81)	0.02
Topcuoglu et al., 2015 [32]	Aerobic exercise 4 weeks	Pain	VAS	VAS Shoulder Daytime: 0.50 ± 1.4 Night: 2.00 ± 2.4 Movement: 4.30 ± 2.2 VAS Hand Daytime hours: 0.45 ± 1.1 Night: 1.40 ± 1.9 Movement: 3.35 ± 2.0	VAS Shoulder Daytime: 2.40 ± 2.7 Night: 2.70 ± 2.5 Movement: 6.00 ± 1.8 VAS Hand Daytime: 2.2 ± 2.3 Night: 2.7 ± 1.9 Movement: 5.4 ± 1.6	0.009 0.376 0.012 0.004 0.041 0.03
		Physical function	Brunnstrom Stage	Not communicated	Not communicated	0.059
		Activities of daily living	FIM	Not communicated	Not communicated	0.001
Barnhoorn et al., 2015 [33]	Pain exposure physiotherapy 5 sessions	Pain	VAS	Start: 6.18 (2.50) 3 months: 4.41 (2.85) 6 months: 4.31 (2.81) 9 months: 3.52 (2.69)	Home: 7.11 (2.01) 3 months: 5.35 (3.09) 6 months: 4.92 (3.34) 9 months: 4.96 (3.02)	0.36
		Physical function	Muscle strength (%) ^+^	Home: 61.90 (22.96) ^++^ 3 months: 36.80 (27.86) ^++^ 6 months: 27.50 (26.52) ^++^ 9 months: 25.83 (27.39) ^++^	Home: 67.14 (23.16) ^++^ 3 months: 46.10 (26.16) ^++^ 6 months: 38.25 (27.20) ^++^ 9 months: 32.50 (27.22) ^++^	0.02
			Active range of motion	Home: 4.71 (2.16) ^++^ 3 months: 3.11 (1.26) ^++^ 6 months: 3.35 (1.67) ^++^ 9 months: 2.89 (1.22) ^++^	Home: 4.93 (1.98) ^++^ 3 months: 4.04 (1.95) ^++^ 6 months: 3.52 (1.26) ^++^ 9 months: 3.32 (0.95) ^++^	0.25
		Activities of daily living	EQ-5D (EuroQol-5D)	Home: 0.53 (0.26) 3 months: 0.63 (0.22) 6 months: 0.77 (0.19) 9 months: 0.76 (0.20)	Home: 0.47 (0.29) 3 months: 0.64 (0.26) 6 months: 0.67 (0.32) 9 months: 0.74 (0.25)	0.79
Christophe et al., 2016 [34]	Prism adaptation 8 sessions	Pain	VAS	Start: 5.88 ± 1.26 Post-treatment: 3.8 ± 0.5	No comparison	<0.0006
		Activities of daily living	SIP	Onset: 38.7 ± 5.76 Post-treatment: 28.6 ± 4.64	No comparison	<0.05
Hollander et al., 2016 [35]	In vivo exposure (EXP) 17 weeks	Pain	NPS	Pretreatment 5.48 (2.22) Post-treatment 2.79 (2.25)	Pretreatment 5.63 (1.63) Post-treatment 4.98 (2.16)	0.001
		Activities of daily living	WAQ	Pretreatment 6.97 (2.48) Post-treatment 1.44 (2.15)	Pretreatment 6.99 (1.76) Post-treatment 4.57 (2.90)	0.054
			RASQ	Pretreatment 3.14 (0.89) Post-treatment 1.86 (0.91)	Pre-treatment 3.49 (0.96) Posttreatment 3.02 (1.19)	<0.001
Schmid et al., 2017 [36]	Sensorimotor training 2 weeks	Pain	VAS	Onset: 4.5 ± 1.52 Post-treatment: 3.5 ± 2.09	No comparison	0.023
Halicka et al., 2021 [19]	PA: Prism adaptation 2 weeks	Pain	NPRS	Home: 5.96 (5.02 to 6.80) Post-treatment: 5.43 (4.55 to 6.24) 3 months: 5.62 (4.69 to 6.48) 6 months: 5.59 (4.69 to 6.41)	Start: 6.15 (5.26 to 7.00) Posttreatment: 5.84 (4.82 to 6.74) 3 months: 6.04 (5.12 to 6.80) 6 months: 5.95 (5.07 to 6.73)	0.126
Machač et al., 2024 [37]	Mirror Therapy 6 weeks	Pain	VAS	Resting Start: 4.1 ± 2.9 42 days: 1.2 ± 0.4 72 days: 0.9 ± 0.1 Movement Onset: 5.9 ± 5.0 42 days: 2.3 ± 1.0 72 days: 2.0 ± 0.7	Rest Onset: 4.4 ± 3.4 42 days: 3.6 ± 2.6 72 days: 2.6 ± 1.6 Movement Onset: 6.7 ± 5.6 42 days 5.6 ± 4.2 72 days: 4.0 ± 3.1	<0.001
			SIP	Baseline: 38.7 ± 5.76 Post-treatment: 28.6 ± 4.64	Home: 37.1 ± 5.89 Post-treatment: 34.2 ± 4.92	*p* < 0.05
Lewis et al., 2021 [38]	Mediated Virtual Reality 4 weeks of intervention + 2 weeks of follow-up	Pain	NPRS	Onset: 5.6 ± 3.0 After 4 weeks: 5.2 ± 2.7 Follow-up at 2 weeks: 4.4 ± 2.5	Onset: 5.7 ± 3.0 After 4 weeks: 5.7 ± 3.0 Follow-up at 2 weeks: 5.7 ± 3.0	*p* < 0.05
Saha et al., 2021 [39]	Mirror Therapy 4 weeks of intervention + 2 weeks of follow-up	Pain	NPRS	Start: 6.07 ± 1.58 Post-treatment: 3.93 ± 1.39 Follow-up at 2 weeks: 3.47 ± 1.30	Start: 6.60 ± 1.06 Post-treatment: 5.33 ± 0.98 Follow-up at 2 weeks: 5.33 ± 1.23	*p* < 0.05
			FIM	Start: 72.47 ± 17.41 Post-treatment: 88.33 ± 18.72 Follow-up at 2 weeks: 92.07 ± 17.21	Start: 62.88 ± 15.40 Post-treatment: 66.38 ± 15.33 Follow-up at 2 weeks: 66.25 ± 15.45	*p* < 0.05

^+^ Muscle strength (%) = left–right difference, relative to the unaffected side; ^++^ A decrease means improvement; DASH: Disability of the Arm, Shoulder and Hand Questionnaire; WMFT: Wolf Motor Function Test; MAL: Motor Activity Record; NPS: neuropathic pain scale; NPRS: Numeric Pain Rating Scale; RASQ: Radboud Skills Questionnaire; WAQ: Walking Ability Questionnaire. SIP: Disease Impact Profile Scale. FIM: Functional Independence Measure. VAS: Visual Analog Scale; EQ-5D is an instrument which evaluates the generic quality of life developed in Europe and widely used; PDI: Pain Disability Index; FMA: The Fugl–Meyer Assessment; SF-MPQ: Short-Form McGill Pain Questionnaire.

**Table 3:** Pain reduction in CRPS by intervention.

Intervention (Studies)	No. of Studies (Design)	Risk of Bias	Inconsistency	Indirectness	Imprecision	Publication Bias	Final Certainty
Mirror therapy (MT) [28,30,37,39]	4 RCTs	Moderate (blinding difficult)	Not serious (consistent effect)	2 in post-stroke/not serious	Small samples	Possible	Moderate
GMI [26,27,29]	2 RCTs + 1 pre–post	Moderate–high	Serious (heterogeneity)	Direct	Small	Possible	Very low
PEPT (exposure) [31,33,35]	3 RCTs	Moderate	Not serious/minor	Direct	Moderate	Possible	Moderate
Prism adaptation [19,34]	1 pilot + 1 RCT	Moderate	Serious (one positive, one null)	Direct	Small	Possible	Low
Virtual reality [38]	1 RCT	Moderate	N/A	Direct	Moderate	Possible	Low
Aerobic exercise [32]	1 RCT	Moderate	N/A	Direct	Small	Possible	Low
Sensorimotor training [36]	1 pilot	High	N/A	Direct	Very small	Probable	Very low

**Table 4:** Physical function.

Intervention (Studies)	No. of Studies (Design)	Risk of Bias	Inconsistency	Indirectness	Imprecision	Publication Bias	Final Certainty
Mirror therapy (MT) [28,30]	2 RCTs	Moderate	Not serious (improvement in WMFT and Fugl–Meyer)	1 in post-stroke/minor	Small	Possible	Moderate
GMI [26,29]	1 RCT + 1 pre–post	High	Serious (heterogeneous)	Direct	Very small	Possible	Very low
PEPT [33,35]	2 RCTs	Moderate	Minor (ROM improved; mixed strength results)	Direct	Moderate	Possible	Moderate
Aerobic exercise [32]	1 RCT	Moderate	N/A	Direct	Small; effect on Brunnstrom unclear	Possible	Very low

**Table 5:** Activities of daily living.

Intervention (Studies)	No. of Studies (Design)	Risk of Bias	Inconsistency	Indirectness	Imprecision	Publication Bias	Final Certainty
Mirror therapy (MT) [28,30,37,39]	4 RCTs	Moderate	Not serious (MAL/FIM/SIP improved)	2 in post-stroke/minor	Small	Possible	Moderate
PEPT (exposure) [31,33,35]	3 RCTs	Moderate	Minor (PDI/EQ-5D/WAQ/RAQ mixed in magnitude)	Direct	Moderate	Possible	Moderate
GMI [29]	1 pre–post (DASH)	High	N/A	Direct	Very small	Possible	Very low
Prism adaptation [34]	1 pilot	Moderate	N/A	Direct	Very small	Possible	Very low
Aerobic exercise [32]	1 RCT	Moderate	N/A	Direct	Small; incomplete reporting	Possible	Very low

## Data Availability

Data exchange not applicable—no new data generated.

## Supplementary Materials

The following supporting information can be downloaded at: https://www.mdpi.com/article/10.3390/jcm14196820/s1, PRISMA 2020 Checklist. Ref. [23] is cited in the Supplementary Materials.

## Appendix A

**Table A1:** Combination of search terms.

1	“Complex Regional Pain Syndromes” AND “Physical Therapy”
2	“Complex Regional Pain Syndromes” AND “Exercise”
3	“Complex Regional Pain Syndromes” AND “Mirror therapy”
4	“Complex Regional Pain Syndromes” AND “Motor imagery”
5	“Complex Regional Pain Syndromes” AND “Pain”
6	“Complex Regional Pain Syndromes” AND “Muscle Strength”
7	“Complex Regional Pain Syndromes” AND “Range of Motion, Articular”
8	“Complex Regional Pain Syndromes” AND “Activities of Daily Living”
9	“Complex Regional Pain Syndromes” AND “Motor function”
10	“Complex Regional Pain Syndromes” AND “cognitive behavioral therapy”
11	“Complex Regional Pain Syndromes” AND “physical therapy modalities”

## Appendix B

**Table A2:** Excluded studies.

	DOI/link	Author, Year	Design/Approach	Reason for Exclusion (According to Criteria)
1	10.1186/1471-2377-4-13	(van de Vusse et al., 2004) [47]	Pharmacological trial	Exclusively pharmacological (no exercise or neurocognitive intervention
2	10.1089/jpm.2022.0539	(Wang et al., 2023) [48]	Case series	Case series—excluded by design.
3	10.1016/j.pain.2009.06.023	(Sigtermans et al., 2009) [49]	Pharmacological RCT	Exclusively pharmacological.
4	10.7326/0003-4819-152-3-201002020-00006	(Goebel et al., 2010) [50]	Pharmacological trial	Exclusively pharmacological.
5	10.1016/j.jbspin.2017.03.009	(Chevreau et al., 2017) [51]	Systematic review/pharmacological meta-analysis	Secondary study and pharmacological; not eligible design/intervention.
6	10.1007/s00482-024-00858-2	(Bernardy et al., 2025) [52]	Review/perspective	Review (not a primary study).
7	10.1097/00002508-199909000-00009	(Sherry et al., 1999) [53]	Pediatric (adolescents)	Pediatric population—excluded.
8	10.1186/s12969-016-0090-8	(Weissmann & Uziel, 2016) [54]	Review (pediatric population)	Review and pediatric—excluded.
9	10.1017/S0266462318000429	(den Hollander et al., 2018) [21]	Economic evaluation (cost-effectiveness)	No therapeutic intervention—excluded.
10	10.1186/s13018-022-03461-2	(Sobeeh et al., 2023) [55]	Systematic review (mechanisms/sensitization)	Not a primary intervention study—excluded.
11	10.1007/s00482-021-00559-0	(Storz & Kraft, 2021) [56]	Summary article/guideline	No estudio primario de intervención excluido.
12	10.3390/medicina57111262	(Moretti et al., 2021) [57]	Scoping review	Review, no primary intervention—excluded.
13	10.3233/BMR-220081	(Kavka, 2023) [58]	Narrative review	Revisión (no elegible por diseño).
14	10.1016/j.jstrokecerebrovasdis.2020.104995	(Altas et al., 2020) [59]	Retrospective observational study (risk factors)	No therapeutic intervention—excluded.
15	10.1080/09638288.2019.1650295	(Mouraux et al., 2021) [60]	Cohort/observational	No therapeutic intervention—excluded.
16	10.1080/09593985.2024.2393213	(Batalla & Lewis, 2025) [61]	Case series	Case series (not RCT/cohort/cross-sectional)—excluded.
17	10.1067/mpd.2002.124380	(Lee et al., 2002) [12]	Pediatric observational study	Pediatric—excluded.
18	10.1097/AJP.0000000000001089	(Shafiee et al., 2023) [62]	Systematic review/meta-analysis	Secondary study (not primary)—excluded.
19	PubMed 24084413	(Oral et al., 2013) [63]	Narrative review	Review, not a primary study—excluded.

## References

[1] Shim H., Rose J., Halle S., Shekane P. (2019). Complex regional pain syndrome: A narrative review for the practising clinician. Br. J. Anaesth..

[2] de Mos M., de Bruijn A.G.J., Huygen F.J.P.M., Dieleman J.P., Stricker C.h.B.H., Sturkenboom M.C.J.M. (2007). The incidence of complex regional pain syndrome: A population-based study. Pain.

[3] Sandroni P., Benrud-Larson L.M., McClelland R.L., Low P.A. (2003). Complex regional pain syndrome type I: Incidence and prevalence in Olmsted county, a population-based study. Pain.

[4] Ott S., Maihöfner C. (2018). Signs and Symptoms in 1,043 Patients with Complex Regional Pain Syndrome. J. Pain.

[5] Goh E.L., Chidambaram S., Ma D. (2017). Complex regional pain syndrome: A recent update. Burn. Trauma.

[6] Rodríguez-López M.J., Fernández-Baena M., Yáñez-Santos J.A. (2014). Complex regional pain syndrome in children: Treatment possibilities. J. Span. Pain Soc..

[7] Hernández-Porras B.C., Plancarte-Sánchez R., Alarcón-Barrios S., Sámano-García M. (2017). Complex regional pain syndrome: A review. Cir. Cir..

[8] Misidou C., Papagoras C. (2019). Complex Regional Pain Syndrome: An update. Mediterr. J. Rheumatol..

[9] Zangrandi A., Allen Demers F., Schneider C. (2021). Complex Regional Pain Syndrome. A Comprehensive Review on Neuroplastic Changes Supporting the Use of Non-invasive Neurostimulation in Clinical Settings. Front. Pain Res..

[10] Forouzanfar T., Köke A.J.A., van Kleef M., Weber W.E.J. (2002). Treatment of complex regional pain syndrome type I. Eur. J. Pain.

[11] Smith T.O. (2005). How effective is physiotherapy in the treatment of complex regional pain syndrome type I? A review of the literature. Musculoskelet. Care.

[12] Lee B.H., Scharff L., Sethna N.F., McCarthy C.F., Scott-Sutherland J., Shea A.M., Sullivan P., Meier P., Zurakowski D., Masek B.J. (2002). Physical therapy and cognitive-behavioral treatment for complex regional pain syndromes. J. Pediatr..

[13] Smart K.M., Wand B.M., O’Connell N.E. (2016). Physiotherapy for pain and disability in adults with complex regional pain syndrome (CRPS) types I and II. Cochrane Database Syst. Rev..

[14] Fenn K., Byrne M. (2013). The key principles of cognitive behavioural therapy. InnovAiT Educ. Inspir. Gen. Pract..

[15] Al Sayegh SAl Filén T., Johansson M., Sandström S., Stiewe G., Butler S. (2013). Mirror therapy for Complex Regional Pain Syndrome (CRPS)-A literature review and an illustrative case report. Scand. J. Pain.

[16] Donati D., Boccolari P., Giorgi F., Berti L., Platano D., Tedeschi R. (2024). Breaking the Cycle of Pain: The Role of Graded Motor Imagery and Mirror Therapy in Complex Regional Pain Syndrome. Biomedicines.

[17] Barnhoorn K.J., Oostendorp R.A.B., van Dongen R.T.M., Klomp F.P., Samwel H., van der Wilt G.J., Adang E., Groenewoud H., van de Meent H., Frölke J.P.M. (2012). The effectiveness and cost evaluation of pain exposure physical therapy and conventional therapy in patients with complex regional pain syndrome type 1. Rationale and design of a randomized controlled trial. BMC Musculoskelet Disord..

[18] Halicka M., Vittersø A.D., Proulx M.J., Bultitude J.H. (2020). Pain reduction by inducing sensory-motor adaptation in Complex Regional Pain Syndrome (CRPS PRISMA): Protocol for a double-blind randomized controlled trial. BMC Neurol..

[19] Halicka M., Vittersø A.D., McCullough H., Goebel A., Heelas L., Proulx M.J., Bultitude J.H. (2021). Prism adaptation treatment for upper-limb complex regional pain syndrome: A double-blind randomized controlled trial. Pain.

[20] Donegan T., Ryan B.E., Sanchez-Vives M.V., Świdrak J. (2022). Altered bodily perceptions in chronic neuropathic pain conditions and implications for treatment using immersive virtual reality. Front. Hum. Neurosci..

[21] den Hollander M., Heijnders N., de Jong J.R., Vlaeyen J.W.S., Smeets R.J.E.M., Goossens M.E.J.B. (2018). In Vivo Exposure Versus Pain-Contingent Physical Therapy in Complex Regional Pain Syndrome Type I: A Cost-Effectiveness Analysis. Int. J. Technol. Assess. Health Care.

[22] Li T.S., Wang R., Su X., Wang X.Q. (2023). Effect and mechanisms of exercise for complex regional pain syndrome. Front. Mol. Neurosci..

[23] Page M.J., McKenzie J.E., Bossuyt P.M., Boutron I., Hoffmann T.C., Mulrow C.D., Shamseer L., Tetzlaff J.M., Akl E.A., Brennan S.E. (2021). The PRISMA 2020 statement: An updated guideline for reporting systematic reviews. BMJ.

[24] Maher C.G., Sherrington C., Herbert R.D., Moseley A.M., Elkins M. (2003). Reliability of the PEDro scale for rating quality of randomized controlled trials. Phys. Ther..

[25] Slim K., Nini E., Forestier D., Kwiatkowski F., Panis Y., Chipponi J. (2003). Methodological index for non-randomized studies (minors): Development and validation of a new instrument. ANZ J. Surg..

[26] Moseley L.G. (2004). Graded motor imagery is effective for long-standing complex regional pain syndrome: A randomised controlled trial. Pain.

[27] Moseley G.L. (2006). Graded motor imagery for pathologic pain. Neurology.

[28] Cacchio A., De Blasis E., De Blasis V., Santilli V., Spacca G. (2009). Mirror Therapy in Complex Regional Pain Syndrome Type 1 of the Upper Limb in Stroke Patients. Neurorehabil. Neural Repair.

[29] Lagueux E., Charest J., Lefrançois-Caron E., Mauger M.E., Mercier E., Savard K., Tousignant-Laflamme Y. (2012). Modified graded motor imagery for complex regional pain syndrome type 1 of the upper extremity in the acute phase. Int. J. Rehabil. Res..

[30] Pervane Vural S., Nakipoglu Yuzer G.F., Sezgin Ozcan D., Demir Ozbudak S., Ozgirgin N. (2016). Effects of Mirror Therapy in Stroke Patients with Complex Regional Pain Syndrome Type 1: A Randomized Controlled Study. Arch. Phys. Med. Rehabil..

[31] Barnhoorn K.J., Staal J.B., van Dongen R.T., Frölke J.P., Klomp F.P., van de Meent H., Samwel H., Nijhuis-van der Sanden M.W. (2015). Are Pain-Related Fears Mediators for Reducing Disability and Pain in Patients with Complex Regional Pain Syndrome Type 1? An Explorative Analysis on Pain Exposure Physical Therapy. PLoS ONE.

[32] Topcuoglu A., Gokkaya N.K.O., Ucan H., Karakuş D. (2015). The effect of upper-extremity aerobic exercise on complex regional pain syndrome type I: A randomized controlled study on subacute stroke. Top. Stroke Rehabil..

[33] Barnhoorn K.J., van de Meent H., van Dongen R.T.M., Klomp F.P., Groenewoud H., Samwel H., der Sanden M.W.G.N.-V., Frölke J.P.M., Staal J.B. (2015). Pain exposure physical therapy (PEPT) compared to conventional treatment in complex regional pain syndrome type 1: A randomised controlled trial. BMJ Open.

[34] Christophe L., Chabanat E., Delporte L., Revol P., Volckmann P., Jacquin-Courtois S., Rossetti Y. (2016). Prisms to Shift Pain Away: Pathophysiological and Therapeutic Exploration of CRPS with Prism Adaptation. Neural. Plast..

[35] den Hollander M., Goossens M., de Jong J., Ruijgrok J., Oosterhof J., Onghena P., Smeets R., Vlaeyen J.W.S. (2016). Expose or protect? A randomized controlled trial of exposure in vivo vs pain-contingent treatment as usual in patients with complex regional pain syndrome type 1. Pain.

[36] Schmid A.C., Schwarz A., Gustin S.M., Greenspan J.D., Hummel F.C., Birbaumer N. (2017). Pain reduction due to novel sensory-motor training in Complex Regional Pain Syndrome I—A pilot study. Scand. J. Pain.

[37] Machač S., Chasáková L., Kakawand S., Kozák J., Štěpánek L., Vejvalka J., Kolář P., Černý R. (2024). Mirror visual feedback as therapeutic modality in unilateral upper extremity complex regional pain syndrome type I: Randomized controlled trial. Eur. J. Phys. Rehabil. Med..

[38] Lewis J.S., Newport R., Taylor G., Smith M., McCabe C.S. (2021). Visual illusions modulate body perception disturbance and pain in Complex Regional Pain Syndrome: A randomized trial. Eur. J. Pain.

[39] Saha S., Sur M., Ray Chaudhuri G., Agarwal S. (2021). Effects of mirror therapy on oedema, pain and functional activities in patients with poststroke shoulder-hand syndrome: A randomized controlled trial. Physiother. Res. Int..

[40] Harden R.N., McCabe C.S., Goebel A., Massey M., Suvar T., Grieve S., Bruehl S. (2022). Complex Regional Pain Syndrome: Practical Diagnostic and Treatment Guidelines, 5th Edition. Pain Med..

[41] Duong S., Bravo D., Todd K.J., Finlayson R.J., Tran D.Q. (2018). Treatment of complex regional pain syndrome: An updated systematic review and narrative synthesis. Can. J. Anesth./J. Can. D’anesthésie.

[42] Garcia-Correa H.R., Sanchez-Montoya L.J., Daza-Arana J.E., Ordoñez-Mora L.T. (2021). Aerobic Physical Exercise for Pain Intensity, Aerobic Capacity, and Quality of Life in Patients with Chronic Pain: A Systematic Review and Meta-Analysis. J. Phys. Act. Health.

[43] Méndez-Rebolledo G., Gatica-Rojas V., Torres-Cueco R., Albornoz-Verdugo M., Guzmán-Muñoz E. (2017). Update on the effects of graded motor imagery and mirror therapy on complex regional pain syndrome type 1: A systematic review. J. Back Musculoskelet. Rehabil..

[44] Schubert C. (2021). Therapie des komplexen regionalen Schmerzsyndroms im Handbereich aus Sicht der Physiotherapie. Unfallchirurg.

[45] Johnson S., Cowell F., Gillespie S., Goebel A. (2022). Complex regional pain syndrome what is the outcome?—A systematic review of the course and impact of CRPS at 12 months from symptom onset and beyond. Eur. J. Pain.

[46] Kraft E., Storz C., Ranker A. (2021). Physikalische Therapie in der Behandlung des komplexen regionalen Schmerzsyndroms. Der Schmerz.

[47] van de Vusse A.C., Stomp-van den Berg S.G., Kessels A.H., Weber W.E. (2004). Randomised controlled trial of gabapentin in Complex Regional Pain Syndrome type 1 [ISRCTN84121379]. BMC Neurol..

[48] Wang A.T., Wang E.J., Smith T.J., Razzak R., Christo P.J. (2023). Scrambler Therapy for Patients with Complex Regional Pain Syndrome: A Case Series. J. Palliat. Med..

[49] Sigtermans M.J., van Hilten J.J., Bauer M.C.R., Arbous S.M., Marinus J., Sarton E.Y., Dahan A. (2009). Ketamine produces effective and long-term pain relief in patients with Complex Regional Pain Syndrome Type 1. Pain.

[50] Goebel A., Baranowski A., Maurer K., Ghiai A., McCabe C., Ambler G. (2010). Intravenous Immunoglobulin Treatment of the Complex Regional Pain Syndrome. Ann. Intern. Med..

[51] Chevreau M., Romand X., Gaudin P., Juvin R., Baillet A. (2017). Bisphosphonates for treatment of Complex Regional Pain Syndrome type 1: A systematic literature review and meta-analysis of randomized controlled trials versus placebo. Jt. Bone Spine.

[52] Bernardy K., Wicking M., Michelka R., Schwarzer A. (2025). Kognitive Verhaltenstherapie beim komplexen regionalen Schmerzsyndrom. Der Schmerz.

[53] Sherry D.D., Wallace C.A., Kelley C., Kidder M., Sapp L. (1999). Short- and Long-term Outcomes of Children with Complex Regional Pain Syndrome Type I Treated with Exercise Therapy. Clin. J. Pain.

[54] Weissmann R., Uziel Y. (2016). Pediatric complex regional pain syndrome: A review. Pediatr. Rheumatol..

[55] Sobeeh M.G., Hassan K.A., da Silva A.G., Youssef E.F., Fayaz N.A., Mohammed M.M. (2023). Pain mechanisms in complex regional pain syndrome: A systematic review and meta-analysis of quantitative sensory testing outcomes. J. Orthop. Surg. Res..

[56] Storz C., Kraft E. (2021). Ergotherapie bei komplexem regionalem Schmerzsyndrom. Der Schmerz.

[57] Moretti A., Palomba A., Paoletta M., Liguori S., Toro G., Iolascon G. (2021). Complex Regional Pain Syndrome in Athletes: Scoping Review. Medicina.

[58] Kavka T. (2023). Harmful or safe? Exposure and pain provocation during physiotherapy of complex regional pain syndrome I: A narrative review. J. Back Musculoskelet. Rehabil..

[59] Altas E.U., Onat Ş.Ş., Konak H.E., Polat C.S. (2020). Post-stroke complex regional pain syndrome and related factors: Experiences from a tertiary rehabilitation center. J. Stroke Cerebrovasc. Dis..

[60] Mouraux D., Lenoir C., Tuna T., Brassinne E., Sobczak S. (2021). The long-term effect of complex regional pain syndrome type 1 on disability and quality of life after foot injury. Disabil. Rehabil..

[61] Batalla M.A.P., Lewis J.S. (2025). Cognitive Multisensory Rehabilitation, a novel approach for Complex Regional Pain Syndrome: Case series. Physiother. Theory Pract..

[62] Shafiee E., MacDermid J., Packham T., Walton D., Grewal R., Farzad M. (2023). The Effectiveness of Rehabilitation Interventions on Pain and Disability for Complex Regional Pain Syndrome. Clin. J. Pain.

[63] Oral A., Ilieva E.M., Küçükdeveci A.A., Varela E., Valero R., Berteanu M., Christodoulou N. (2013). Generalised and regional soft tissue pain syndromes. The role of physical and rehabilitation medicine physicians. The European perspective based on the best evidence. A paper by the UEMS-PRM Section Professional Practice Committee. Eur. J. Phys. Rehabil. Med..

